# Role of phage ϕ1 in two strains of *Salmonella* Rissen, sensitive and resistant to phage ϕ1

**DOI:** 10.1186/s12866-018-1360-z

**Published:** 2018-12-07

**Authors:** Marina Papaianni, Felice Contaldi, Andrea Fulgione, Sheridan L. Woo, Angela Casillo, Maria Michela Corsaro, Ermenegilda Parrilli, Luca Marcolungo, Marzia Rossato, Massimo Delledonne, Marianna Garonzi, Domenico Iannelli, Rosanna Capparelli

**Affiliations:** 1 Department of Agriculture, University of Naples “Federico II”, via Università 100, 80055 Portici, Naples Italy; 2 Department of Pharmacy, University of Naples “Federico II”, via Domenico Montesano 49, 80131 Naples, Italy; 3 National Research Council, Institute for Sustainable Plant Protection, via Università 133, 80055 Portici, Naples Italy; 4 Task Force on Microbiome Studies, University of Naples “Federico II”, via Università 100, 80055 Portici, Naples Italy; 5 Department of Chemical Sciences, University of Naples “Federico II”, via Cintia 4, 80126 Naples, Italy; 6 Department of Biotechnology, University of Verona, Strada Le Grazie 15, 37134 Verona, Italy; 7 Personal Genomics, Strada le Grazie 15, 37134 Verona, Italy

**Keywords:** Phage selection, *Salmonella* Rissen, Cost of resistance, Phase variation, Repeatable evolution

## Abstract

**Background:**

The study describes the *Salmonella* Rissen phage ϕ1 isolated from the ϕ1-sensitive *Salmonella* Rissen strain R^W^. The same phage was then used to select the resistant strain R^R^ϕ1+, which can harbour or not ϕ1.

**Results:**

Following this approach, we found that ϕ1, upon excision from R^W^ cells with mitomycin, behaves as a temperate phage: lyses host cells and generates phage particles; instead, upon spontaneous excision from R^R^ϕ1+ cells, it does not generate phage particles; causes loss of phage resistance; switches the O-antigen from the smooth to the rough phenotype, and favors the transition of *Salmonella* Rissen from the planktonic to the biofilm growth.

The R^W^ and R^R^ϕ1+ strains differ by 10 genes; of these, only two (phosphomannomutase_1 and phosphomannomutase_2; both involved in the mannose synthesis pathway) display significant differences at the expression levels. This result suggests that phage resistance is associated with these two genes.

**Conclusions:**

Phage ϕ1 displays the unusual property of behaving as template as well as lytic phage. This feature was used by the phage to modulate several phases of *Salmonella* Rissen lifestyle.

**Electronic supplementary material:**

The online version of this article (10.1186/s12866-018-1360-z) contains supplementary material, which is available to authorized users.

## Background

Bacteria are under constant attack by bacteriophages (phages), the most abundant life forms in the biosphere [1]. They have evolved a variety of defense mechanisms against phages, which in turn have evolved mechanisms to offset the defense plans set up by bacteria [2]. Generally, phages recognize only very few strains of the same bacterial species [3], a tactic maximizing the benefits from recombination with phages having the same lifestyle and genomic organization [4]. Bacteria frequently gain resistance by losing the phage receptor [2] or reducing its binding specificity [5]. Bacteria can also promote a temporary change of the phage receptor specificity. They do it through a mechanism known as phase variation. In a context of antagonistic co-evolution [6], rapidity in the response to a phage attack is fundamental for bacterial survival. Phase variation confers resistance at a much faster rate than random mutation [7]. Bacteria and phages both exploit phase variation: *S. enterica* ser. Typhimurium to express alternative forms of the O-antigen and escape phage attack [8, 9]; *Escherichia* (*E.*) *coli* phage Mu [10] and other phages [11] to alternatively express different ligands and expand their host range. Phage receptors often function also as bacterial virulence factors. The reversibility of phase variation curbs this toll by limiting it strictly to the duration of phage infection. In addition to reversibility, phase variation displays the property of regulating the expression of several traits in a co-ordinate fashion [8], a feature that adds efficiency to this mechanism. Recent studies show that - to maximize survival of a fraction of the population in case of sudden environmental changes - reversible phase variation can occur randomly [12]. In conclusion, the above examples well explain how the role of phase variation in the bacterial world is to rapidly generate diversity and enable bacteria to colonize different hosts and survive in changing environments [8].

The term superinfection exclusion (SE) describes the property of a preexisting prophage to inhibit a secondary infection by the same – or a very close – phage [13, 14]. SE is mediated by proteins that block the penetration of phage DNA inside the host cell soon after infection [2]. As an example, the SE protein A of *S. enterica* ser. Typhimurium carrying the lysogenic phage P22 confers protection against infection by the phages L, MG178, or MG40 [15]. The proteins blocking the phage DNA penetration can be of bacterial or phage origin. SE in fact can benefit the phage as well as the host. SE, reducing the cost of phage infection, sets conditions for a mutualistic relationship [16], where the phage benefits of increased transmission opportunities and protection against predators, while providing the host with virulence factors [17], toxins [18], or promoting gene transfer and thus bacterial genome variability [19].

Here we describe the *S.* Rissen phage ϕ1. This phage was excised from the ϕ1-sensitive *S.* Rissen strain R^W^ and then used to select the ϕ1-resistant strain R^R^ϕ1+, which can spontaneously lose ϕ1. The ϕ1 excised from R^W^ cells with mitomycin behaves as an inducible temperate phage since lyses host cells and generates phage particles. Instead, the spontaneous excision of ϕ1 from R^R^ϕ1+ cells does not generate phage particles, promotes biofilm production, loss of phage resistance, and the switch of the O-antigen from smooth to rough. To carry out the above tasks, phage ϕ1 uses all the resources described earlier: phase variation, SE, and SE inhibition.

Finally, because of their rapid evolution and easy replication of experiments, bacteria are frequently used to investigate whether evolution is contingent or repeatable, an issue still debated [20]. Here we show that four independent ϕ1-resistant clones isolated from the same ϕ1-sensitive strain R^W^, all display identical mutations at two *phosphomannomutase* genes.

## Results

### Phage isolation and bacterial strains characterization

**Fig. 1:**
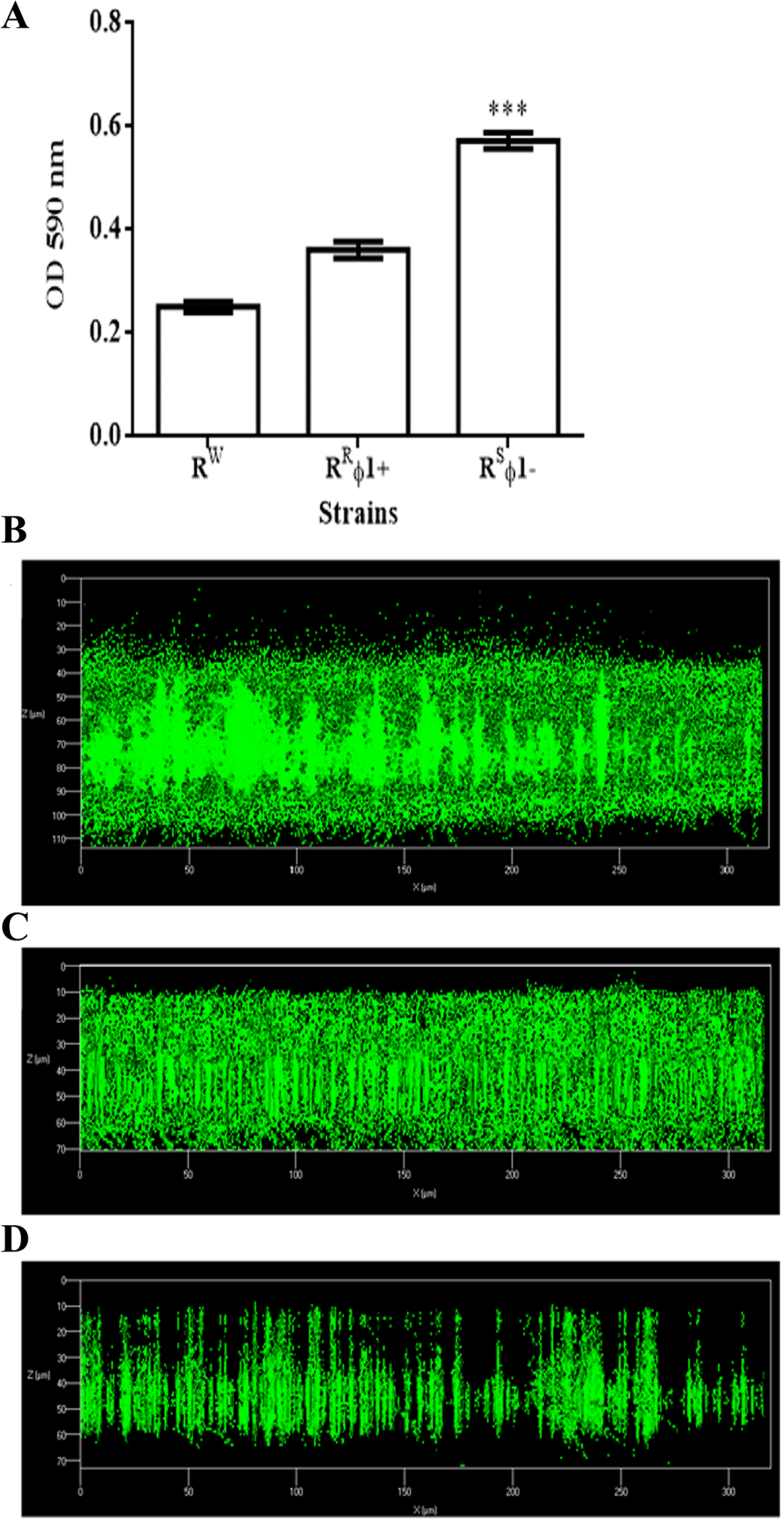
Phage ϕ1 influences biofilm production. **a** Quantitative and **b-d** Confocal Laser Scanning Microscopy (CLSM) analyses of biofilm produced by: **b** R^S^ϕ1-; **c** R^R^ϕ1+; and **d** R^W^ bacteria. Bacteria were grown in 8-well chamber slide for 20 h and then stained with LIVE/DEAD reagents. Green fluorescence (SYTO9) indicates viable cells and red fluorescence (PI) dead cells

Following incubation with mitomycin C, the wild type *S.* Rissen bacteria (R^W^) yielded phage ϕ1 (titer: 10^7^ PFU/mL; burst size: 50 PFU/cell) and release of ϕ1 particles was followed by host cell lysis. Instead, the spontaneous release of ϕ1 from R^R^ϕ1+ cells (R^S^ϕ1-) occurs without recovery of phage particles and is also associated with increased biofilm production (Fig. 1a-d) and the phage-sensitive phenotype (Table 1). Ordinarily, lysogenic strains are immune to the phage that they produce (phenomenon known as SE). Remarkably, R^W^ bacteria were positive by the double layer agar (DLA) method with ϕ1, indicating that the ϕ1 prophage is resistant to the SE mechanism.

**Table 1:** Distinctive traits of the different *Salmonella* Rissen strains

Strain	Biofilm production	Φ1 presence	Morphology phenotype	Φ1 resistance
R^W^	+/−	+	*pdar*	–
R^R^ϕ1+	++	+	*ras*	+
R^S^ϕ1-	++++	–	*pdar*	–

1–4: Amount of Biofilm production; +: positive; −: negative; *pdar* Pink dry and red phenotype, *ras* Red and smooth phenotype

**Fig. 2:**
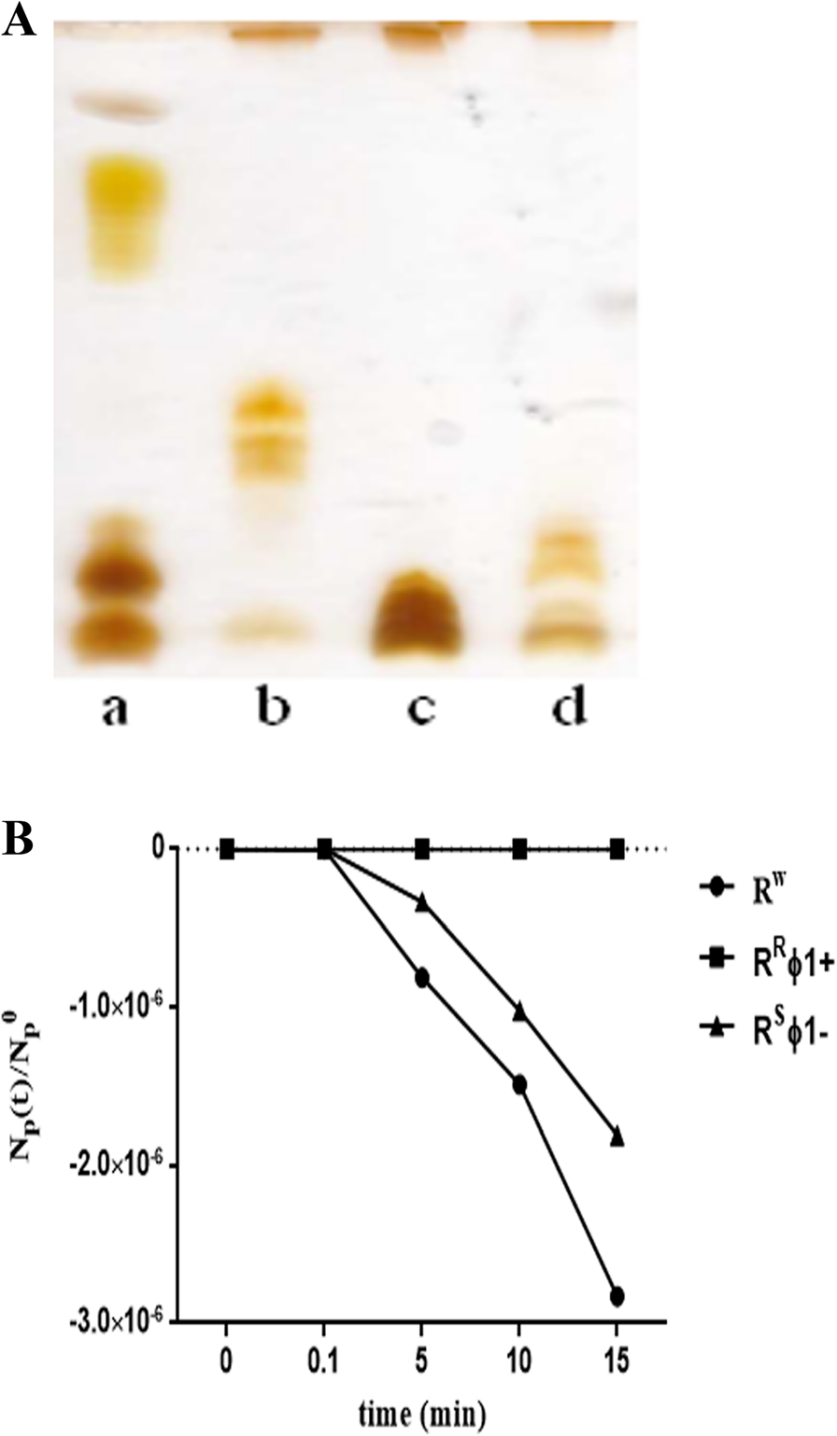
Strains chemical phenotypes and phage ϕ1 binding activities. **a** R^R^ϕ1+ (lane b), R^S^ϕ1- (lane c), and R^W^ (lane d) display the smooth, rough, and semi-rough phenotypes, respectively. The reference marker (lane a) is the LPS from *E. coli* O55:B5. **b** R^R^ϕ1+ strain (ϕ1-resistant and smooth) does not bind the phage; R^W^ strain (ϕ1-sensitive and semi-rough) binds the phage, while R^S^ϕ1- strain (ϕ1-sensitive and rough) displays an intermediate level of phage binding activity

Moreover, R^R^ϕ1+ and R^W^ colonies differ in curli production: R^R^ϕ1+ colonies express the “*ras*” (red and smooth) phenotype, while R^W^ colonies display the “*pdar*” (pink red and dry) phenotype (characterized by a reduced amount of curli) [21] (Table 1). Furthermore, the strains R^S^ϕ1- and R^W^ are both ϕ1-sensitive, while that R^R^ϕ1+ is ϕ1-resistant (Table 1). Also, DOC- polyacrylamide gel electrophoresis of lipopolysaccharide (LPS) showed that the ϕ1-sensitive strains R^W^ and R^S^ϕ1- display the semi-rough and rough phenotypes, respectively, while the ϕ1-resistant strain R^R^ϕ1+ shows the smooth phenotype (Fig. 2a). Accurate phage ϕ1 adsorption experiments confirmed that ϕ1 binds to the semi-rough or rough strains but not to the smooth one (Fig. 2b). Phages specific for rough strains have already been described in *S. enterica* ser. Typhimurium [22, 23] and *Pseudomonas (P.) aeruginosa* [24]. Further, carbohydrate analysis of LPS indicated that ϕ1-sensitive cells - compared to the ϕ1-resistant ones - are associated with higher mannose synthesis (Additional file 1: Figure S1).

### Genome sequencing and assembling

**Fig. 3:**
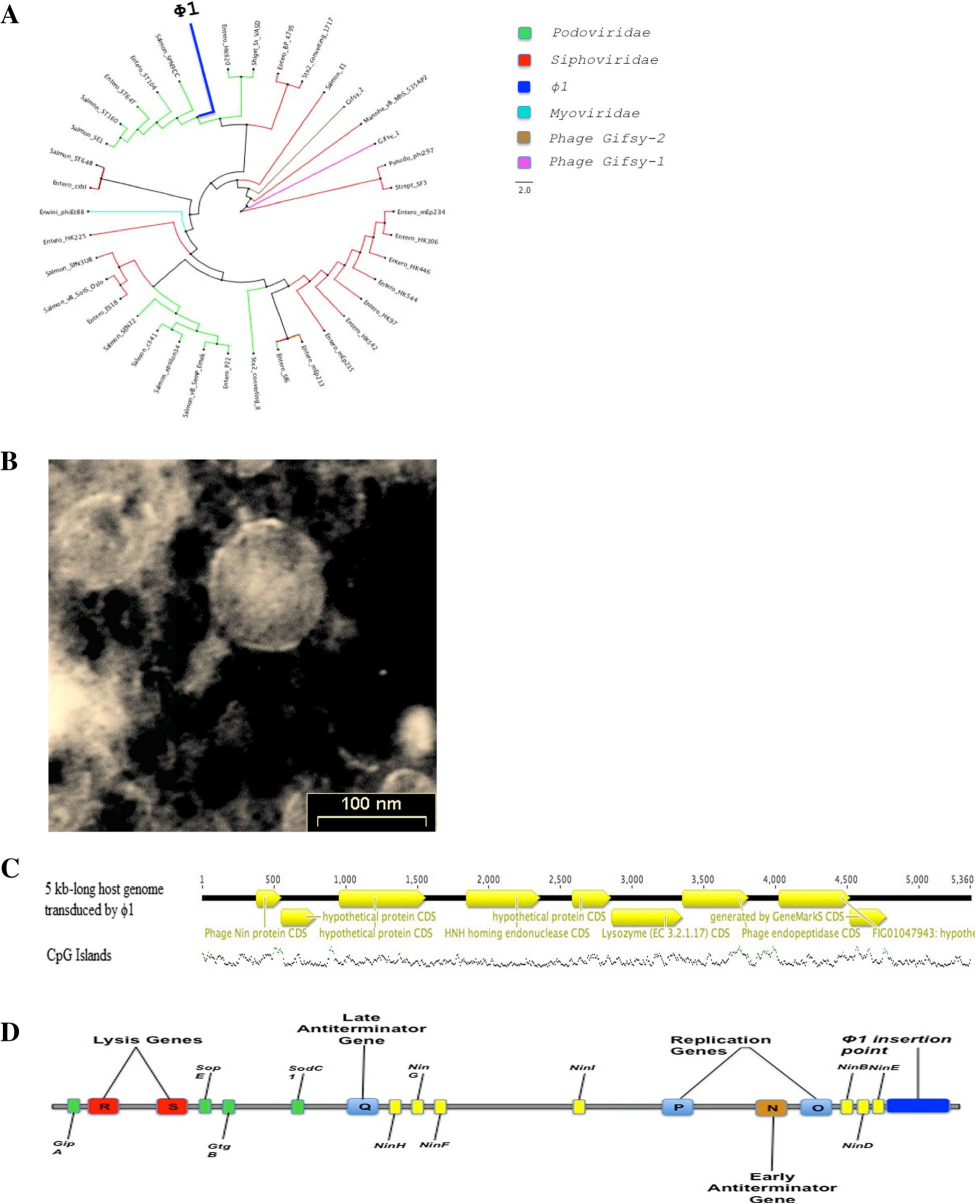
Phage ϕ1 properties. **a** The phylogenetic tree shows a strong DNA identity between ϕ1 and 5 members of the Podovoridae (3 Salmon and 2 Entero phages). The tree is based on the alignment of 39 phage genomes. The bar indicates branch length scale. **b** Transmission Electron Microscopy (TEM) of bacteriophage ϕ1. The short and non-contractile tail, characteristic of Podoviridae, confirms ϕ1 as a member of this family. The scale bar corresponds to 100 nm. **c** Upon excision, phage ϕ1 transduces a 5 kb long region of the host genome. The region includes the following genes: 5 hypothetical proteins, 1 phage endopeptidase, 1 HNH homing endonuclease, 1 lysozyme and 1 phage Nin protein. **d** The phage ϕ1 insertion point is at the end of the R^R^ϕ1+ strain genome (from 4,828,664 to 4, 834, 023 bps)

Phage ϕ1 yielded a total of 2,199,543 reads (660 Mb) and an average coverage of 13,200 x. The de-novo assembled phage ϕ1 genome is 51,738 bp long with a GC content of 48,4%. The genome contains 87 predicted coding sequences (CDSs): 30 affecting bacteriophage physiology, 12 encoding phage structures, 10 regulating DNA replication, and 3 encoding bacterial lysis. Genome sequence and general phage organization can be found in the annotation (available on GenBank accession: KY709687). The phylogenetic tree of phage ϕ1 genome was reconstructed by comparing its proteome with those of 37 fully sequenced phage genomes. Phage ϕ1 disclosed a robust orthology with 5 members of *Podovoridae* (3 *Salmon* and 2 *Entero phages*: 53–72% DNA identity) and therefore assigned to this family (Fig. 3a). The short, stubby, and non-contractible tail confirmed ϕ1 as a member of the *Podoviridae* family (Fig. 3b). Data generated from the R^R^ϕ1+ bacteria by PacBio sequencing evidenced that the phage is circular and double-stranded. Upon mitomycin-induced excision, ϕ1 transduces a 5 kb-long portion of the host genome from R^R^ϕ1+, and R^W^ (Fig. 3c). Apparently, transduction of the 5 kb fragment occurs randomly (Additional file 2: Figure S2).

Illumina sequencing of R^W^ and R^R^ϕ1+ was generated 8,036,355 (2.4 Gb; coverage 602x) and 12,639,370 reads (3.8 Gb; coverage 948x), respectively. To identify the insertion site of ϕ1, we assembled de novo the reads generated from R^W^ and R^R^ϕ1+ and compared them. This approach yielded 104,974 reads with an average length of 5305 bp for a total of 556 Mb and an average coverage of 116X. We could thus establish that in the R^R^ϕ1+ strain phage ϕ1 is inserted at the end of the genome (from 4,828,664 to 4,834,023 bps) (Fig. 3d).

### Phage resistance results from frameshift mutations in two genes of the mannose pathway

**Fig. 4:**
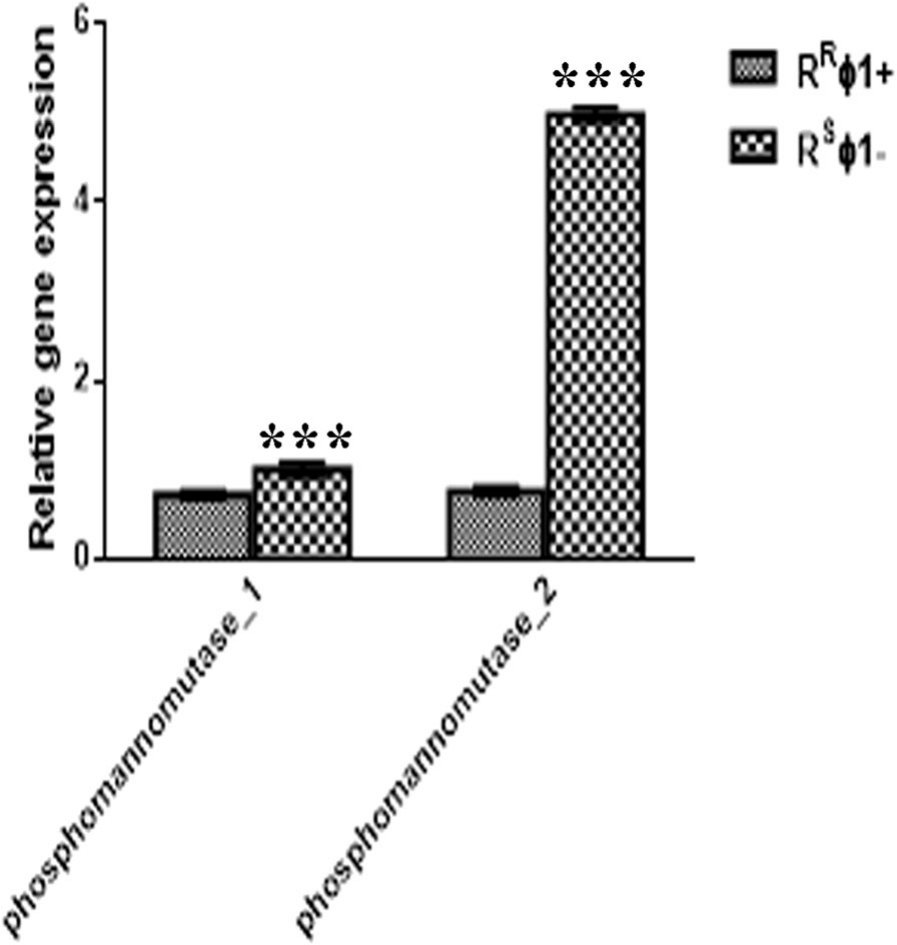
Differences in expression levels of the 10 genes differentiating the R^R^ϕ1+ and R^S^ϕ1- strains. The resistant strain (R^R^ϕ1+) displays significantly reduced expression levels of the *phosphomannomutase1* and *phosphomannomutase2* genes, compared to the sensitive strain (R^S^ϕ1-). The relative gene expression levels are expressed using the R^W^ strain as internal comparison

**Fig. 5:**
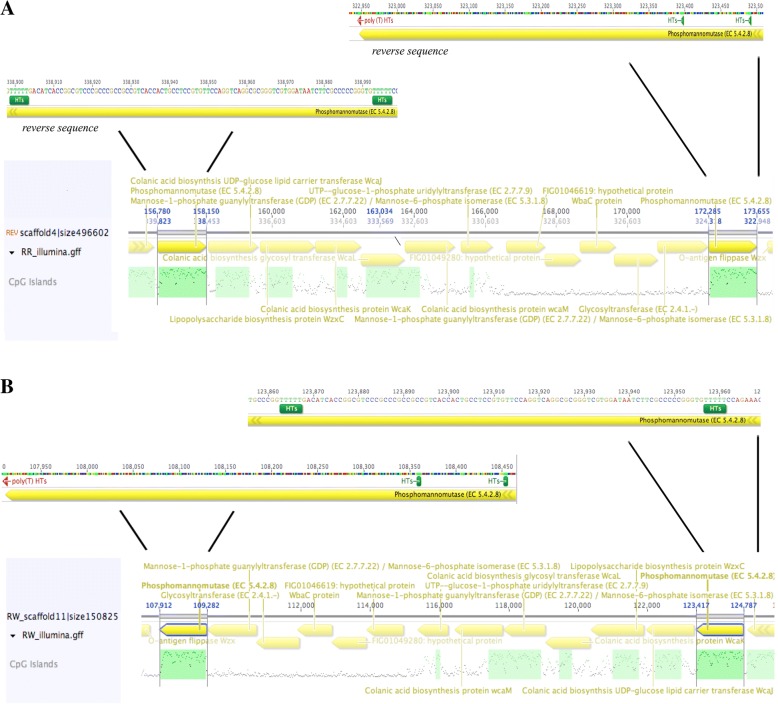
Map of homopolymeric tracts (HTs) in the *Phosphomannomutase* genes. Localization of HTs within the coding region is indicated in green at poly(A) and in red at poly(T). **a** and **b** indicate HTs localization in R^R^ɸ1+ and R^W^ respectively

Comparative genomics showed that the two strains R^W^ and R^R^ϕ1+ differ by 10 genes, each displaying from 1 to 15 SNP sites (Additional file 3: Table S1). The expression levels of the genes *phosphomannomutase1* and *phosphomannomutase2* participating to the mannose synthesis are higher in the susceptible strain R^S^ϕ1- compared to the resistant strain R^R^ϕ1+ (Fig. 4). This result concurs with evidence from carbohydrate analysis of LPS (Additional file 1: Figure S1). We conclude that phage ϕ1 resistance is associated with reduced expression levels of the *phosphomannomutase1* and *phosphomannomutase2* genes. As often observed in bacteria [25–27], phage ϕ1 resistance was gained by phase variation via frameshift mutation in homopolymeric tracts (HTs) (Fig. 5a and b). Four independent phage-resistant mutants from R^W^ (R^R1–4^) all displayed the same differential gene expression already observed in the original strains R^W^ and R^R^ ϕ1 + .

## Discussion

This study describes the properties of ϕ1, a prophage which modulates several phases of *S.* Rissen life style. In general, prophages aid bacteria with the production of virulence molecules [18], toxins [28], antibiotics [18], or (as in this study) support the bacterial host conferring phage resistance (Fig. 2b), increasing biofilm production (Fig. 1a-d), and providing new genetic material (Fig. 5a and b and Additional file 3: Table S1).

Given the importance of ϕ1 in the life style of the *S.* Rissen, it seems plausible to suggest that the absence of superinfection immunity serves to permit ϕ1 to rapidly abandon or re-infect the host, as environmental circumstances require.

We found that induction of ϕ1 excision with mitomycin in R^W^ cells leads to replication and release of phage particles. Instead, ϕ1 excision from R^R^ϕ1+ cells - spontaneous or induced by thermal shock – does not lead to replication and release of phage particles. This result suggests that phage replication is inhibited in R^R^ϕ1+ cells. As already proposed for the *Listeria (L.) monocytogenes* ϕ10403S, we speculate that ϕ1 or the R^R^ϕ1+ host cells can disconnect phage excision from phage replication and release [29].

The cases of bacterial genes controlled by prophage excision generally involve cryptic prophages [30, 31]. Phage ϕ1 is not cryptic since, following induction with mitomycin, produces infective particles. Thus, ϕ1 is a rare - but not unique [31] – example of non-cryptic prophage influencing the expression of the host cell genes.

Many bacterial species, including *Salmonella*, gain phage resistance by altering the genes of the LPS biosynthesis pathway [24]. In *E. coli*, nine different genes are involved in the LPS biosynthesis pathway, which potentially could lead to T7 phage resistance, but bacteria reach resistance by altering *waaG*, the gene associated with reduced biological cost [32].

Our study describes similar results. Four independent ϕ1-resistant strains isolated from the same strain (R^W^) and grown under the same conditions displayed the same regulatory alteration at two genes (*phosphomannomutase1* and p*hosphomannomutase2*) (Fig. 4). Parallel evolution has also been reported in *L. monocytogenes* [33], *E. coli* [34] and *Propionibacterium* (*P.) acnes* [35]. These results suggest that whenever it is possible, phage resistance is acquired using the path requiring a lower cost. The same explanation could be extended to the acquisition of phage resistance by phase variation, as observed in several bacterial species: *Campylobacter (C.) jejuni* [25], *Vibrio* (*V.) cholerae* [26], *L. monocytogenes* [27, 33], *Herpes* (*H.) influenzae* [36], *Staphylococcus* (*S.) aureus* [37], and *S.* Rissen (this study). Also, in most of these bacterial species (including *S.* Rissen), phase variation originates from HTs frameshift mutations (Fig. 5a and b) and is reversible. Phage resistance by frameshift mutations instead is rapid and reversible: once phage infection ends, the phage-resistant bacteria can revert to the more adaptive phage-sensitive genotype.

## Conclusions

This study describes a phage which modulates several properties of its host. The results of this study may stimulate researchers to better understand benefits and negative outcomes associated with the therapeutic use of phages; how the stability of mutations is influenced by environmental stresses; how phages affect evolution and pathogenicity of bacteria. Finally, the study demonstrates that, at least in bacteria, natural selection uses repeatedly the same evolutionary path, when it requires a lower biological cost.

## Methods

### Bacterial strains

The *S.* Rissen strain R^W^ (serotype 6; antisera were from Staten Serum, Copenhagen, Denmark) was isolated from a food matrix and characterized by Istituto Zooprofilattico Sperimentale Del Mezzogiorno (Portici, Naples, Italy). The *S.* Rissen strain R^R^ was derived - in the course of this research - from the R^W^ strain following selection for resistance to phage ϕ1 as described in this study. R^R^ cells can spontaneously lose the prophage and thus occur with (R^R^ϕ1+) or without ϕ1 (R^S^ϕ1-) (the superscript S indicates that loss of ϕ1 causes loss of phage resistance). All the bacterial strains were analyzed for cellulose production and LPS phenotype and stored at − 20 °C in LB (Sigma-Aldrich, Milan, Italy) supplemented with glycerol (10%; Sigma-Aldrich, Milan, Italy).

### Isolation of the phage ϕ1

Phage ϕ1 excision was induced by incubating R^W^ cells (2 × 10^8^ CFU/5 mL) in LB broth containing 1 μg/mL mitomycin C (Sigma-Aldrich, Milan, Italy) for 1 h at 37 °C. Following centrifugation (5.7 × 10^3^ g), the supernatant was stored at + 4 °C, and the pellet resuspended in 5 mL of LB broth and incubated again at 37 °C for 4 h and then centrifuged. The pellet was discarded, while the supernatants from the two centrifugations were pooled and filtered (filter pore size: 0.22 μm; MF-Millipore, Darmstadt, Germany) [38].

The titer of phage, expressed as plaque forming units (PFU), was evaluated by using the DLA technique as reported by Sambrook et al. [39] Phage ϕ1 was stored in SM buffer at − 20 °C. The aliquot in use was kept at + 4 °C.

### Isolation of the phage ϕ1-resistant strain R^R^

R^W^ bacteria in early exponential growth phase were mixed with warm soft agar. The mixture was poured on LB agar (Sigma-Aldrich, Milan, Italy) plates and allowed to solidify. Phage ϕ1 was then spotted (10 μL/spot) and the plates were incubated overnight at 37 °C. The following morning, the colonies grown inside the lysis plaques were picked with a sterile loop and streaked on LB agar plate. This procedure was repeated 3 times. Phage-resistant bacteria were further tested for phage ϕ1 resistance by the spot test. Plaque absence after overnight incubation confirmed that bacteria were phage ϕ1-resistant (R^R^ϕ1+). Phage ϕ1-resistant colonies were detected after about 24 h of selection.

### Lysogenization

R^S^ϕ1- bacteria (10^8^ CFU in 500 μL LB) were incubated with ϕ1 isolated from R^W^ bacteria (10^8^ PFU/mL) for 72 h. The suspension was mixed with soft agar (4 mL) and then poured on a solid agar. Phage ϕ1was spotted on soft agar (10 μL/spot) and plates were incubated at + 37 °C and inspected daily for plaque formation.

### Analysis of cellulose production

Cellulose production was detected by growing bacteria on LB agar supplemented with 200 μg/mL calcofluor (Sigma-Aldrich, Milan, Italy). Plates were incubated at 37 °C for 2–4 days. Colonies were visualized under a 366-nm light source [40]. Congo red binding was detected by growing bacteria on LB agar supplemented with Congo red (40 μg/mL; Sigma-Aldrich, Milan, Italy).

### Biofilm thickness determined by confocal laser scanning microscopy

Biofilms were formed on polystyrene Chamber Slides (n° 177,445; Thermo Scientific, Ottawa, Canada). For this purpose, overnight cultures of R^R^ϕ1+, R^S^ϕ1-, and R^W^ strains grown in LB medium were diluted to a final concentration of 0.001 and seeded into a chamber slide at 37 °C for 36 h to assess biofilm thickness and cell viability. The biofilm cell viability was determined with the FilmTracer™ LIVE/DEAD® Biofilm Viability Kit (Molecular Probes, Invitrogen, Carlsbad, California, USA) following the manufacturer’s instructions. Microscopic observations and image acquisitions were performed as described [41].

### Salmonella genome sequencing, assembly and annotation

The R^W^ or R^R^ strains were expanded in LB broth starting from a single colony. Genomic DNA was then extracted by the phenol-chloroform method, purified with Agencourt AMPure XP beads (Beckman Coulter; beads to DNA ratio 1.8:1), and quantified by the Qubit dsDNA BR Assay Kit (Thermo Fisher, MA, USA). DNA size and purity were measured by the 2200 Tape Station Instrument (Agilent Genomics) and Nanodrop (Thermo Fisher), respectively. Illumina libraries were obtained from 1 μg of genomic DNA, and sequenced with the NextSeq500 instrument using the 150 nt paired-end protocol (Illumina, San Diego, CA). Illumina reads were quality filtered, trimmed using Sickle and finally quality corrected with BayesHammer. Genomes were assembled de novo from Illumina reads using SPAdes 2.9.0 with multiple k-mer combinations: from 101 to 125 with 2 nt steps for the 202 R^R^ genome, with 95, 97, 111, 113 for the R^W^ genome and 101, 105, 109, 113, 117, 121,125 for the ϕ1 genome. The resultant contigs were scaffolded using SSPACE 3.0. Five μg high-molecular-weight genomic DNA (peak >60Kb) were used to prepare ~ 20 Kb-insert SMRT-bell libraries (Pacific Biosciences, CA, USA). The library templates were sequenced using the single molecule real time (SMRT) Sequencing technology on a PacBio RSII sequencer (Pacific Biosciences, Macrogen Inc., Korea). PacBio subreads were extracted using Bash5tools (version 0.8.0), filtered and assembled de-novo with Falcon-Integrate and the settings suggested for bacterial genome. The assembled genome sequence was polished by Quiver v 0.9.2. and gene annotation performed using RAST web service (http://rast.nmpdr.org/) [42]. The ϕ1 insertion site was identified by mapping PacBio reads from R^R^ϕ1+ bacteria against the phage genome assembly and soft-clipped bases were retrieved.

### Variant SNP calling

SNP calling was carried out using MUMmer 3.23 tool [43]. Single-nucleotide polymorphisms (SNPs) were identified by Show-SNPs, a script associated with MUMmer 3.23.

The output was filtered by BUFF > 50 with the Show- SNPs flags ClIrx 25 and the SNP position was assembly by quality > 80. The R^W^ *Salmonella* genome was used as reference. Each assembly was queried with each SNP context from the MUMmer output using BLAST + [44], retaining only SNPs for which exactly one occurrence of either of the two genomes was found in all assemblies.

### Chemical analysis

PAGE was performed using the system of Laemmli [45] with sodium deoxycholate (DOC; Thermo Scientific, Waltham, MA USA) as detergent as described [46]. Glycosyl analysis was performed as reported [47].

### Real time PCR

Total RNA was extracted from individual bacterial strains according to the Allprep Bacterial DNA/RNA/Protein Kit protocol (Quiagen) and then reverse-transcribed using the high-capacity cDNA reverse transcription kit (Applied Biosystem). Real-time PCR was carried out using the Step One Real-Time PCR Systems machine (Thermo-Fisher scientific). Reactions were carried out in a 20 μl of Master SYBR Green I mix (Roche Diagnostics Ltd., Lewes, UK). The amplification protocol included 10 min at 95 °C and 40 cycles, each consisting of 10 s at 95 °C for denaturation, 120 min at 57 °C for annealing, and 60 s at 60 °C for extension; the final step was at 4 °C. PCR reactions were carried out in triplicate. Expression values were normalized versus the R^W^ strain. The reference gene was the housekeeping *InvA*. The relative gene expression was carried out using the Delta Delta ct Method [48].

### Other methods

Following the genome sequencing experiment, we designed the primers for the 5 kb region using primer 3 as primer design tool. We used the following overlapping eight primer pairs. Primers are designed to amplify regions within a size range of 400–600 bp.

The thermal shock of R^W^ or R^R^ϕ1+ cells was carried out by exposing the cells at − 20 °C for 1 h and + 40 °C for 2 h. The cells were then tested for loss of phage resistance. The phylogenetic tree was constructed using the maximum likelihood method [49]; for data alignment were used the Blosum 65 (gap open penalty = 11; gap extension penalty = 3), Jukes-Cantor, and UPGMA models. Biofilm production was measured by the crystal violet assay [50].

## Additional files

Additional file 1: **Figure S1.** Gas chromatography-mass spectrometry (GC-MS) analysis of the (A) R^W^, (B) R^R^ϕ1+, and (C) R^S^ϕ1-strains. All of the strains display the presence of glucose, glucosamine, heptose, and KDO. Acquisition of phage resistance by R^R^ϕ1+ strain is associated with loss of mannose. Peaks marked with X represent methyl esters of fatty acids. (PDF 673 kb)

Additional file 2: **Figure S2.** Electrophoresis gel of PCR for detecting the presence of 5 kb region. Lines 1–5: RWϕ1+; lines 6–9: RWϕ1-; lines 10–14: RRϕ1+; lines 15–18: RSϕ1-; M = marker (100 kb). (PDF 35 kb)

Additional file 3: **Table S1.** SNPs detection analysis of R^W^ and R^R^ɸ1+ strains. (PDF 107 kb)

## Abbreviations

CDSs: Coding sequences
DLA: Double layer agar
HTs: Homopolymeric tracts
LPS: Lipopolysaccharide
Pdar: Pink red and dry
Ras: Red and smooth
SE: Superinfection exclusion
