# Supplementary figures and images for: Role of phage ϕ1 in two strains of Salmonella Rissen, sensitive and resistant to phage ϕ1
Source: BMC Microbiol. 2018 Dec 7;18:208. doi: 10.1186/s12866-018-1360-z (PMC6286511; doi:10.1186/s12866-018-1360-z)

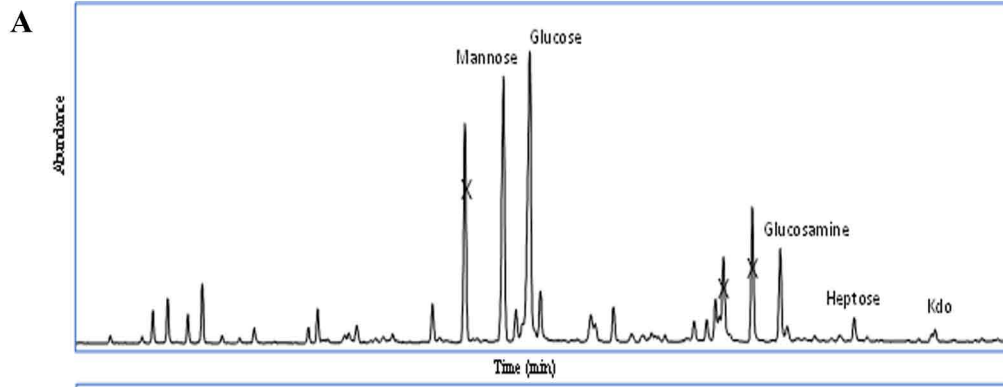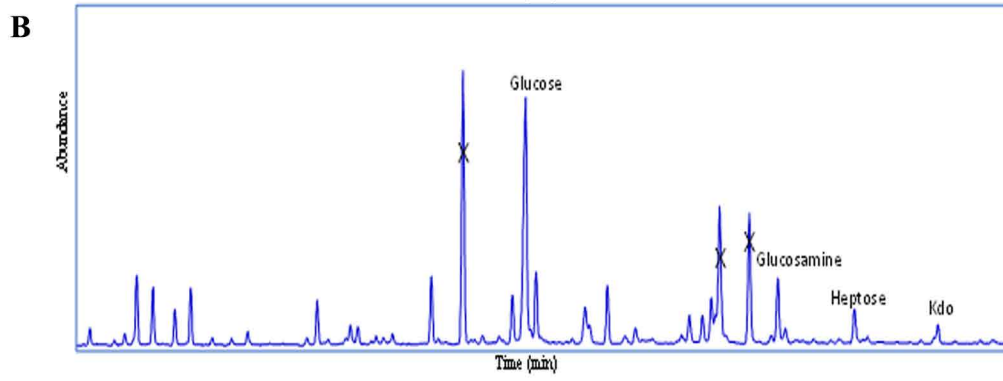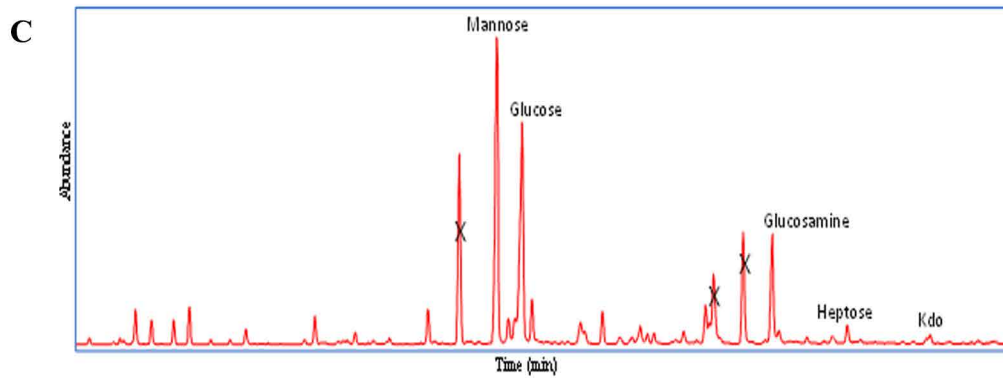

Supplement: Supplementary file 1 — Figure S1. Gas chromatography-mass spectrometry (GC-MS) analysis of the (A) RW, (B) RRϕ1+, and (C) RSϕ1-strains. All of the strains display the presence of glucose, glucosamine, heptose, and KDO. Acquisition of phage resistance by RRϕ1+ strain is associated with loss of mannose. Peaks marked with X represent methyl esters of fatty acids. (PDF 673 kb) [file 12866_2018_1360_MOESM1_ESM.pdf]

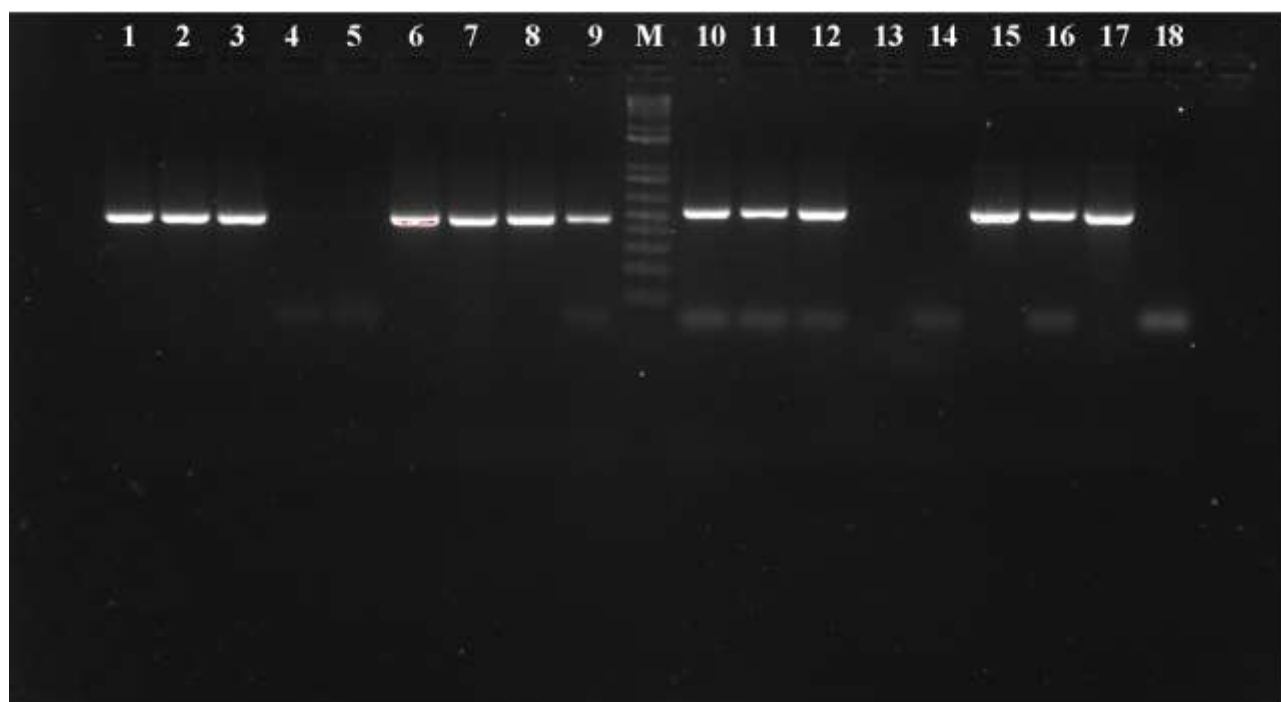

Supplement: Supplementary file 2 — Figure S2. Electrophoresis gel of PCR for detecting the presence of 5 kb region. Lines 1–5: RWϕ1+; lines 6–9: RWϕ1-; lines 10–14: RRϕ1+; lines 15–18: RSϕ1-; M = marker (100 kb). (PDF 35 kb) [file 12866_2018_1360_MOESM2_ESM.pdf]
