# Supplementary material for: Role of phage ϕ1 in two strains of Salmonella Rissen, sensitive and resistant to phage ϕ1
Source: BMC Microbiol. 2018 Dec 7;18:208. doi: 10.1186/s12866-018-1360-z (PMC6286511; doi:10.1186/s12866-018-1360-z)
Supplement: Supplementary file 3 — Table S1. SNPs detection analysis of RW and RRɸ1+ strains. (PDF 107 kb) [file 12866_2018_1360_MOESM3_ESM.pdf]

Additional file 3: SNPs detection analysis carried out on R^W and $R^R\phi1+$ strains.

<table>
<tr><th>Gene Name</th><th>R^W Position</th><th>SUB</th><th>R^Rϕ1+ Position</th><th>BUFF</th><th>DIST</th><th>FRM</th><th>TAGS</th></tr>
<tr><td>AIDA autotransporter-like protein</td><td>115184</td><td>C T</td><td>69161</td><td>3849</td><td>69118</td><td>1 -1</td><td>RW_scaffold10|size184301 scaffold9|size184356</td></tr>
<tr><td>putative MR-MLE-family protein</td><td>162871</td><td>C A</td><td>21397</td><td>25</td><td>21397</td><td>1 -1</td><td>RW_scaffold10|size184301 scaffold9|size184356</td></tr>
<tr><td>Large Subunit Ribosomal RNA%3B lsuRNA%3B LSU rRNA</td><td>211</td><td>T C</td><td>1889</td><td>15</td><td>70</td><td>1 1</td><td>RW_scaffold117|size280 scaffold26|size2428</td></tr>
<tr><td>Large Subunit Ribosomal RNA%3B lsuRNA%3B LSU rRNA</td><td>226</td><td>A G</td><td>1904</td><td>15</td><td>55</td><td>1 1</td><td>RW_scaffold117|size280 scaffold26|size2428</td></tr>
<tr><td>Large Subunit Ribosomal RNA%3B lsuRNA%3B LSU rRNA</td><td>270</td><td>G T</td><td>1948</td><td>5</td><td>11</td><td>1 1</td><td>RW_scaffold117|size280 scaffold26|size2428</td></tr>
<tr><td>Large Subunit Ribosomal RNA%3B lsuRNA%3B LSU rRNA</td><td>275</td><td>C A</td><td>1953</td><td>5</td><td>6</td><td>1 1</td><td>RW_scaffold117|size280 scaffold26|size2428</td></tr>
<tr><td>Phosphomannomutase (EC 5.4.2.8)</td><td>108191</td><td>C T</td><td>323227</td><td>622</td><td>42635</td><td>1 1</td><td>RW_scaffold11|size150825 scaffold4|size496602</td></tr>
<tr><td>Phosphomannomutase (EC 5.4.2.8)</td><td>108813</td><td>T C</td><td>323849</td><td>622</td><td>42013</td><td>1 1</td><td>RW_scaffold11|size150825 scaffold4|size496602</td></tr>
<tr><td>Phosphomannomutase (EC 5.4.2.8)</td><td>123783</td><td>G A</td><td>338819</td><td>27</td><td>27043</td><td>1 1</td><td>RW_scaffold11|size150825 scaffold4|size496602</td></tr>
<tr><td>Phosphomannomutase (EC 5.4.2.8)</td><td>123810</td><td>A G</td><td>338846</td><td>27</td><td>27016</td><td>1 1</td><td>RW_scaffold11|size150825 scaffold4|size496602</td></tr>
<tr><td>Phosphomannomutase (EC 5.4.2.8)</td><td>124318</td><td>T C</td><td>339354</td><td>508</td><td>26508</td><td>1 1</td><td>RW_scaffold11|size150825 scaffold4|size496602</td></tr>
<tr><td>Cell division protein FtsK</td><td>121666</td><td>G A</td><td>777216</td><td>8311</td><td>8755</td><td>1 -1</td><td>RW_scaffold14|size130420</td></tr>
</table>

									scaffold3|size900164
Dihydropteroate synthase (EC 2.5.1.15)	652267	A	G	101900	1	101900	1	-1	RW_scaffold1|size754294 scaffold2|size701334
Dihydropteroate synthase (EC 2.5.1.15)	652268	G	T	101899	1	101899	1	-1	RW_scaffold1|size754294 scaffold2|size701334
Dihydropteroate synthase (EC 2.5.1.15)	652269	C	G	101898	1	101898	1	-1	RW_scaffold1|size754294 scaffold2|size701334
Dihydropteroate synthase (EC 2.5.1.15)	652271	C	G	101896	1	101896	1	-1	RW_scaffold1|size754294 scaffold2|size701334
Dihydropteroate synthase (EC 2.5.1.15)	652272	T	C	101895	1	101895	1	-1	RW_scaffold1|size754294 scaffold2|size701334
Dihydropteroate synthase (EC 2.5.1.15)	652279	A	G	101888	1	101888	1	-1	RW_scaffold1|size754294 scaffold2|size701334
Dihydropteroate synthase (EC 2.5.1.15)	652280	C	A	101887	1	101887	1	-1	RW_scaffold1|size754294 scaffold2|size701334
Cytochrome c heme lyase subunit CcmF	2818	G	A	2915	615	1274	1	1	RW_scaffold29|size4556 scaffold23|size4188
Large Subunit Ribosomal RNA%3B lsuRNA%3B LSU rRNA	1765	T	A	268	38	42	1	-1	RW_scaffold34|size2227 scaffold95|size309
putative surface-exposed virulence protein	30312	A	C	30534	1	30312	1	1	RW_scaffold4|size421594 scaffold5|size422018
putative surface-exposed virulence protein	30313	T	G	30535	1	30313	1	1	RW_scaffold4|size421594 scaffold5|size422018
putative surface-exposed virulence protein	30314	C	G	30536	1	30314	1	1	RW_scaffold4|size421594 scaffold5|size422018

putative surface-exposed virulence protein	30315	G	C	30537	1	30315	1	1	RW_scaffold4|size421594 scaffold5|size422018
putative surface-exposed virulence protein	30318	A	T	30540	3	30318	1	1	RW_scaffold4|size421594 scaffold5|size422018
putative surface-exposed virulence protein	30324	T	A	30545	3	30324	1	1	RW_scaffold4|size421594 scaffold5|size422018
putative surface-exposed virulence protein	30327	T	A	30548	1	30327	1	1	RW_scaffold4|size421594 scaffold5|size422018
putative surface-exposed virulence protein	30328	G	C	30549	1	30328	1	1	RW_scaffold4|size421594 scaffold5|size422018
putative surface-exposed virulence protein	30329	T	A	30550	1	30329	1	1	RW_scaffold4|size421594 scaffold5|size422018
putative surface-exposed virulence protein	30330	A	C	30551	1	30330	1	1	RW_scaffold4|size421594 scaffold5|size422018
putative surface-exposed virulence protein	30332	G	C	30554	1	30332	1	1	RW_scaffold4|size421594 scaffold5|size422018
putative surface-exposed virulence protein	30333	G	C	30555	1	30333	1	1	RW_scaffold4|size421594 scaffold5|size422018
putative surface-exposed virulence protein	30334	A	C	30556	1	30334	1	1	RW_scaffold4|size421594 scaffold5|size422018
putative surface-exposed virulence protein	30335	A	C	30557	1	30335	1	1	RW_scaffold4|size421594 scaffold5|size422018
putative surface-exposed virulence protein	30336	A	G	30558	1	30336	1	1	RW_scaffold4|size421594 scaffold5|size422018
DamX2C an inner membrane protein	39817	T	A	40042	1	39817	1	1	RW_scaffold4|size421594

involved in bile resistance									scaffold5|size422018
DamX2C an inner membrane protein involved in bile resistance	39818	G	A	40043	1	39818	1	1	RW_scaffold4|size421594 scaffold5|size422018
DamX2C an inner membrane protein involved in bile resistance	39820	T	A	40045	1	39820	1	1	RW_scaffold4|size421594 scaffold5|size422018
DamX2C an inner membrane protein involved in bile resistance	39828	T	G	40053	2	39828	1	1	RW_scaffold4|size421594 scaffold5|size422018
DamX2C an inner membrane protein involved in bile resistance	39830	C	A	40055	2	39830	1	1	RW_scaffold4|size421594 scaffold5|size422018
DamX2C an inner membrane protein involved in bile resistance	39837	G	A	40059	1	39837	1	1	RW_scaffold4|size421594 scaffold5|size422018
DamX2C an inner membrane protein involved in bile resistance	39838	C	A	40060	1	39838	1	1	RW_scaffold4|size421594 scaffold5|size422018
DamX2C an inner membrane protein involved in bile resistance	39839	G	A	40061	1	39839	1	1	RW_scaffold4|size421594 scaffold5|size422018
Alcohol dehydrogenase (EC 1.1.1.1)%3B Acetaldehyde dehydrogenase (EC 1.2.1.10)	251	C	T	899914	21	251	1	-1	RW_scaffold7|size216118 scaffold3|size900164
Alcohol dehydrogenase (EC 1.1.1.1)%3B Acetaldehyde dehydrogenase (EC 1.2.1.10)	272	G	C	899893	21	272	1	-1	RW_scaffold7|size216118 scaffold3|size900164
Alcohol dehydrogenase (EC 1.1.1.1)%3B Acetaldehyde dehydrogenase (EC 1.2.1.10)	419	T	G	899746	39	419	1	-1	RW_scaffold7|size216118 scaffold3|size900164
Alcohol dehydrogenase (EC 1.1.1.1)%3B Acetaldehyde dehydrogenase (EC 1.2.1.10)	458	C	T	899707	36	458	1	-1	RW_scaffold7|size216118 scaffold3|size900164

Alcohol dehydrogenase (EC 1.1.1.1)%3B Acetaldehyde dehydrogenase (EC 1.2.1.10)	494	A	C	899671	21	494	1	-1	RW_scaffold7|size216118 scaffold3|size900164
Alcohol dehydrogenase (EC 1.1.1.1)%3B Acetaldehyde dehydrogenase (EC 1.2.1.10)	515	T	C	899650	6	515	1	-1	RW_scaffold7|size216118 scaffold3|size900164
Alcohol dehydrogenase (EC 1.1.1.1)%3B Acetaldehyde dehydrogenase (EC 1.2.1.10)	521	C	T	899644	6	521	1	-1	RW_scaffold7|size216118 scaffold3|size900164
Alcohol dehydrogenase (EC 1.1.1.1)%3B Acetaldehyde dehydrogenase (EC 1.2.1.10)	527	A	G	899638	6	527	1	-1	RW_scaffold7|size216118 scaffold3|size900164
Alcohol dehydrogenase (EC 1.1.1.1)%3B Acetaldehyde dehydrogenase (EC 1.2.1.10)	1076	C	T	899089	176	1076	1	-1	RW_scaffold7|size216118 scaffold3|size900164
Small Subunit Ribosomal RNA%3B ssuRNA%3B SSU rRNA	252	C	T	988	18	41	1	1	RW_scaffold99|size292 scaffold32|size1391
Small Subunit Ribosomal RNA%3B ssuRNA%3B SSU rRNA	270	A	C	1006	18	23	1	1	RW_scaffold99|size292 scaffold32|size1391
TolA protein	156679	A	G	43868	3949	37782	1	-1	RW_scaffold9|size194460 scaffold8|size229310

SUB character or gap position of the reference and query sequence respectively;

BUFF distance from the SNP to the nearest mismatch (end of alignment, indel, SNP, etc) in the same alignment;

DIST distance from this SNP to the nearest sequence;

FRM sequence direction or reading frame;

TAGS the reference and query FastA IDs respectively;
